# Jmjd6a regulates *GSK3β* RNA splicing in *Xenopus laevis* eye development

**DOI:** 10.1371/journal.pone.0219800

**Published:** 2019-07-30

**Authors:** Jee Yoon Shin, Jeongin Son, Won Sun Kim, Jungsug Gwak, Bong-Gun Ju

**Affiliations:** Department of Life Science, Sogang University, Seoul, Korea; University of Colorado Boulder, UNITED STATES

## Abstract

It has been suggested that Jmjd6 plays an important role in gene regulation through its demethylation or hydroxylation activity on histone and transcription factors. In addition, Jmjd6 has been shown to regulate RNA splicing by interaction with splicing factors. In this study, we demonstrated that *Jmjd6a* is expressed in developing *Xenopus laevis* eye during optic vesicle formation and retinal layer differentiation stages. Knockdown of *Jmjd6a* by an antisense morpholino resulted in eye malformation including a deformed retinal layer and no lens formation. We further found down-regulation of gene expression related to eye development such as *Rx1*, *Otx2*, and *Pax6* in *Jmjd6a* morpholino injected embryos. Jmjd6 interacts with splicing factor U2AF25 and *GSK3β* RNA in the anterior region of *Xenopus* embryos. Knockdown of *Jmjd6a* led to deletion of *GSK3β* RNA exon 1 and 2, which resulted in generation of N’-terminal truncated GSK3β protein. This event further caused decreased phosphorylation of β-catenin and subsequently increased β-catenin stability. Therefore, our result may suggest that Jmjd6a plays an important role in *Xenopus* eye development through regulation of *GSK3β* RNA splicing and canonical Wnt/β-catenin signaling.

## Introduction

During vertebrate eye development, spatiotemporal orchestration of eye-specific gene expression occurs between different cell types from different embryonic origins including the neural ectoderm, surface ectoderm, and periocular mesenchyme [1]. Eye development is initiated at the onset of gastrulation by the determination of the eye field in the anterior neuroectoderm [2]. As gastrulation proceeds, the eye field is expanded by proliferation and the retinal primordium is formed. Evagination of the neuroectoderm forms optic vesicles and subsequently the optic vesicles are transformed into optic cups with retinal differentiation [3]. Ocular development begins with the formation of the optic vesicles, which come into close contact with the surface ectoderm where the lens placode is formed. The optic cups are further subdivided into the outer retinal pigment epithelium (RPE), the neural retina, and the ciliary marginal zone (CMZ), which is located at the edge of the retina and contains a proliferative population of undifferentiated cells such as retinal stem and progenitor cells. After maturation, the neural retina forms three well-structured layers consisting of the outer nuclear layer (ONL), the inner nuclear layer (INL), and the ganglion cell layer (GCL). The ONL, which is the outermost retinal layer, contains the cell bodies of cone and rod photoreceptors. In the INL, the cell bodies of bipolar, horizontal, and amacrine interneurons exist. The GCL, which is the innermost retinal layer, contains the cell bodies of ganglion cells. Müller glial cells span all retinal layers. These developmental events are tightly regulated by signaling cascades, which control cellular proliferation and differentiation [1].

Among cellular signaling pathways, the canonical Wnt/β-catenin signaling pathway plays an essential role in eye development [4–6]. Wnt/β-catenin signaling is initiated by binding of Wnt to the Frizzled/LRP5/6 receptor, which leads to the accumulation and nuclear translocation of β-catenin. In the nucleus, β-catenin interacts with transcription factors and regulates the expression of their target genes. In the absence of Wnt, β-catenin is phosphorylated at Thr 41, Ser 33, Ser 37, and Thr 41 by GSK3β, which is a member of the β-catenin destruction complex composed of multiple proteins including APC, axin, and CK1α. This subsequently triggers β-catenin destabilization by ubiquitination [7, 8]. In vertebrate eye development, Wnt/β-catenin signaling is activated in distinctive regions of the optic vesicle and the optic cup for dorsoventral patterning [9]. It is subsequently restricted to the peripheral RPE [5]. Wnt/β-catenin signaling is also activated in the CMZ to maintain the population of retinal progenitors [10].

Jumonji domain-containing protein 6 (Jmjd6) (previously known as phosphatidylserine receptor) is a member of the large family of JmjC domain-containing metalloenzymes. Jmjd6 is an iron- and 2–oxoglutarate-dependent dioxygenase that catalyzes the demethylation or hydroxylation of various proteins [11, 12]. For example, Jmjd6 demethylates histone H3 at arginine 2 and histone H4 at arginine 3 [13], estrogen receptor (ERα) [14], RNA helicase A [15], Pax3 [16], and TRAF6 [17]. Recently, the tyrosine kinase activity of Jmjd6 on histone H2A.X has been reported [18]. Also, the role of Jmjd6 in RNA splicing has been reported [19–23]. Jmjd6 catalyzes hydroxylation of splicing factor U2AF65 and regulates alternative RNA splicing [19]. In addition, Jmjd6 plays important roles in multiple biological processes including cell differentiation, proliferation, migration, and apoptosis [24–26]. Therefore, knockdown or knockout of *Jmjd6* demonstrates multiple defects in the developing brain, heart, notochord, and somite in zebrafish, frog, and mouse [27–29].

In this study, we demonstrated that *Jmjd6a* is expressed highly in the eye and brain originated from anterior neural tissue during *Xenopus* development. Knockdown of *Jmjd6a* by antisense morpholino led to augmented canonical Wnt/β-catenin signaling through generation of aberrant *GSK3β* RNA, which resulted in the production of N-terminal truncated GSK3β protein. In turn, these events may cause abnormal eye development in *Xenopus* embryos.

## Materials and methods

### Animal manipulation

*Xenopus laevis* fertilized eggs were dejellied in 2% cysteine (pH 8.0) and grown in 0.1×MMR (Marc’s Modified Ringer). Developmental stages of embryo were determined in accordance with Nieuwkoop and Faber’s staging system [30]. Animals were handled in accordance with a standard protocol approved by the animal care committee of Sogang University.

### RT-PCR

Total RNA was extracted from *Xenopus* embryos using RNAiso Plus (Takara). First-strand cDNA was synthesized from 5μg of total RNA with PrimeScript RT Master Mix (Takara). For real time-PCR, the resulting cDNAs were amplified using 2×PreMIX SYBR kit (Enzynomics) with Stratagen Mx3000p (Agilent Technologies). PCR conditions entailed an initial denaturation at 95°C for 10 min followed by 40 cycles of denaturation (95°C for 15 sec), annealing (60°C for 40 sec) and elongation (72°C for 60 sec), with a final elongation at 72°C for 10 min. Expression was calculated from the cycle threshold (Ct) value using the ΔCt method for quantification. Expression of *EF1α* was used for normalization. For conventional PCR, cDNAs were amplified with GeneAmp PCR system 2700 (Applied Biosystems). PCR conditions entailed an initial denaturation at 95°C for 2 min followed by 33 cycles of denaturation (95°C for 30 sec), annealing (60°C for 30 sec) and elongation (72°C for 30 sec), with a final elongation at 72°C for 5 min. Expression of *EF1α* was used for normalization. Oligonucleotides used for RT- PCR are listed in Supporting Information (S1 Table).

### Whole-mount *in situ* hybridization, histology, and immunohistochemistry

For whole-mount *in situ* hybridization, *Xenopus* embryos were fixed in MEMFA (3.7% formaldehyde, 100 mM MOPS, 2 mM EGTA, 1 mM MgSO_4_) for overnight at 4°C. After serial dehydration in ethanol, the embryos were processed to whole-mount *in situ* hybridization in accordance with the standard protocol with minor modifications [31]. Antisense probes were transcribed by T7 or SP6 RNA polymerase using linearized pGEM T-easy plasmid vector containing *Jmjd6a*, *Jmjd6b*, *Otx2*, *Rx1*, or *Pax6* cDNA with DIG-labeling kit (Boehringer Mannheim). Oligonucleotides used for subcloning are listed in Supporting Information (S1 Table). Hybridization was carried out with 1 μg/ml of riboprobes in reaction solution [50% formamide, 5× SSC (pH 4.5), 1% SDS, 50 μg/ml yeast tRNA, and 50 μg/ml heparin] at 70°C for overnight. The hybridized embryos were washed consecutively with 5X SSC, 2X SSC, and 0.2X SSC containing 50% formamide for 15 min at 62°C. Anti-DIG antibody (Roche) was incubated with the hybridized embryos in PBST solution (0.1% Tween 20 in PBS) for overnight at 4°C. Color reaction was carried out using BM purple alkaline phosphatase substrate (Boehringer Mannheim). The color reaction was terminated with stop solution (1mM EDTA, 0.1% Tween 20 in PBS). For histology, embryos were fixed in MEMFA (3.7% formaldehyde, 100 mM MOPS, 2 mM EGTA, 1 mM MgSO_4_) for overnight at 4°C, dehydrated in ethanol, embedded in paraffin, and sectioned at 7 μm. The tissue sections were deparaffinized and stained with hematoxylin and eosin. For immunohistochemistry, paraffin embedded embryos were sectioned at 5 μm and dried for overnight. After deparaffinization of tissue sections, antigen retrieval was performed with sodium citrate buffer (10mM sodium citrate, 0.05% Tween 20, pH 6.0). Tissue sections were immunostained with anti-Jmjd6 (LS-C30343, LSbio, 1:200) and anti-Islet1 (AB_1157901, Developmental Studies Hybridoma Bank, 1:50) antibodies. TRITC-conjugated goat anti-mouse IgG (T5393, Sigma‐Aldrich) or TRITC-conjugated goat anti-rabbit IgG (T2402, Sigma-Aldrich) was used as the secondary antibody.

### Injection of morpholino antisense oligonucleotide and RNA

*Jmjd6a* morpholino antisense oligonucleotide (*Jmjd6a* MO, 5’-ATCCCCGGTGTTTCC TGACCACCTC-3’) against the *Xenopus laevis Jmjd6a* (NM_001092479) was designed and synthesized (Gene Tools). For negative control, 5’-mismatched oligonucleotide of *Jmjd6a* MO (Control MO, 5’-ATGCCCCGTGTTTGCTGAGCAGCTC-3’) was synthesized (Gene Tools). Morpholino (16 ng) was injected into one or two animal-dorsal blastomeres of 8-cell stage embryos. To test the specificity of *Jmjd6a* MO, 10 ng of synthesized *Jmjd6a* mRNA without 5’-UTR (Δ5’UTR *Jmjd6a*) was co-injected into 2-cell stage embryos. For translation of *GSK3β* RNA, 5 ng of synthesized *GSK3β* mRNA without exon 1 and 2 was injected into 2-cell stage embryos.

### 5’ RACE and RNA synthesis

To identify the 5’ end of *Xenopus GSK3β* RNA, 5’ RACE was performed with mRNA extracted from *Jmjd6a* MO injected embryos using 5’-full RACE core kit (Takara). The cDNAs were subcloned into pGEM T-easy plasmid vector. After sequencing, *GSK3β* cDNA without exon 1 and 2 was subcloned into pCS4+ plasmid vector. *Xenopus Jmjd6a* cDNA without 5’ UTR (Δ5’UTR *Jmjd6a*) was subcloned into pSC2+ basic plasmid vector. For *in vitro* transcription, capped mRNA was synthesized using SP6 mMESSAGE mMACHINE Kit (AM2075, Ambion). Oligonucleotides used for 5’ RACE and subcloning are listed in Supporting Information (S1 Table).

### Immunoprecipitation and western blot analysis

Embryos were homogenized in RIPA buffer [10 mM Tris-Cl (pH 8.0), 1 mM EDTA, 1% Triton X-100, 0.1% sodium deoxycholate, 0.1% SDS, 140 mM NaCl] in the presence of complete protease inhibitors (Roche) and 1mM phenylmethylsulphonylfluoride. Yolk was removed by centrifugation for 10 min at 4°C. The extract was incubated with anti-Jmjd6 antibody (LS-C30343, LSbio) for overnight at 4°C and subsequently incubated with protein A/G agarose beads (Sigma‐Aldrich) for 4 hr at 4°C. The beads were washed extensively and mixed with SDS sample buffer. For negative control, rabbit IgG (12–370, Millipore) was used. Western blotting was performed with anti-Jmjd6 (LS-C30343, LSbio, 1:1000), anti-α/β tubulin (2148, Cell Signaling, 1:1000), anti-U2AF65 (ab151582, Abcam, 1:1000), anti-β-catenin (610154, BD Transduction Laboratories, 1:1000), anti-phospho-β-catenin (Ser33/37/Thr41) (9561S, Cell Signaling Technology, 1:1000), and anti-GSK3β (SC-81462, SC-71186, Santa Cruz Biotechnology, 1:1000) antibodies.

### RNA-immunoprecipitation

RNA-immunoprecipitation was performed in accordance with the standard protocols with minor modifications [32, 33]. Embryos were cross-linked with 1% formaldehyde in PBS and incubated in ice-cold nuclear isolation buffer [0.32 M sucrose, 10 mM Tris-HCl (pH 7.5), 5 mM MgCl_2_, 1% Triton X-100] for 20 min with frequent mixing. After centrifugation for 15 min at 2500g, the resulting nuclei pallet was incubated with RIP buffer [150 mM KCl, 25 mM Tris (pH 7.4), 5 mM EDTA, 0.5 mM DTT, 0.5% NP40, 120 U/ml RNase inhibitor, protease inhibitor cocktail]. Anti-Jmjd6 antibody (LS-C30343, LSbio) or rabbit IgG (12–370, Millipore) coupled to protein G agarose bead (SC-2002, Santa Cruz Biotechnology) was incubated with the nuclear extracts for overnight at 4°C. After washing the beads with ice-cold RIP buffer, DNase I (2270A, Takara) was treated. The RNAs were extracted with phenol/chloroform and precipitated by ammonium acetate/ethanol. Oligonucleotides are listed in Supporting Information (S1 Table).

### TOP-Flash assay

TOP-Flash reporter plasmid (50 pg) containing multiple copies of Tcf-binding site was injected with CMV-Renilla luciferase plasmid (5 pg) into two blastomeres of the animal-dorsal region at the 8-cell stage. The anterior regions of the injected embryos were collected at stage 26 and a luciferase assay was performed using the Dual-Luciferase Assay System (Promega). Renilla luciferase activity served as an internal control for normalizing the firefly luciferase activity.

### Statistical analyses

All quantitative data are presented as mean ± standard deviation (SD). for three independent experiments. For examination of gene expression, more than 30 embryos were analyzed in each experiment. The differences between two groups were evaluated by a paired t-test. Significance values were *P ≤ 0.05 and **P ≤ 0.01.

## Result

### Expression pattern of *Jmjd6* in *Xenopus laevis* development

**Fig 1:**
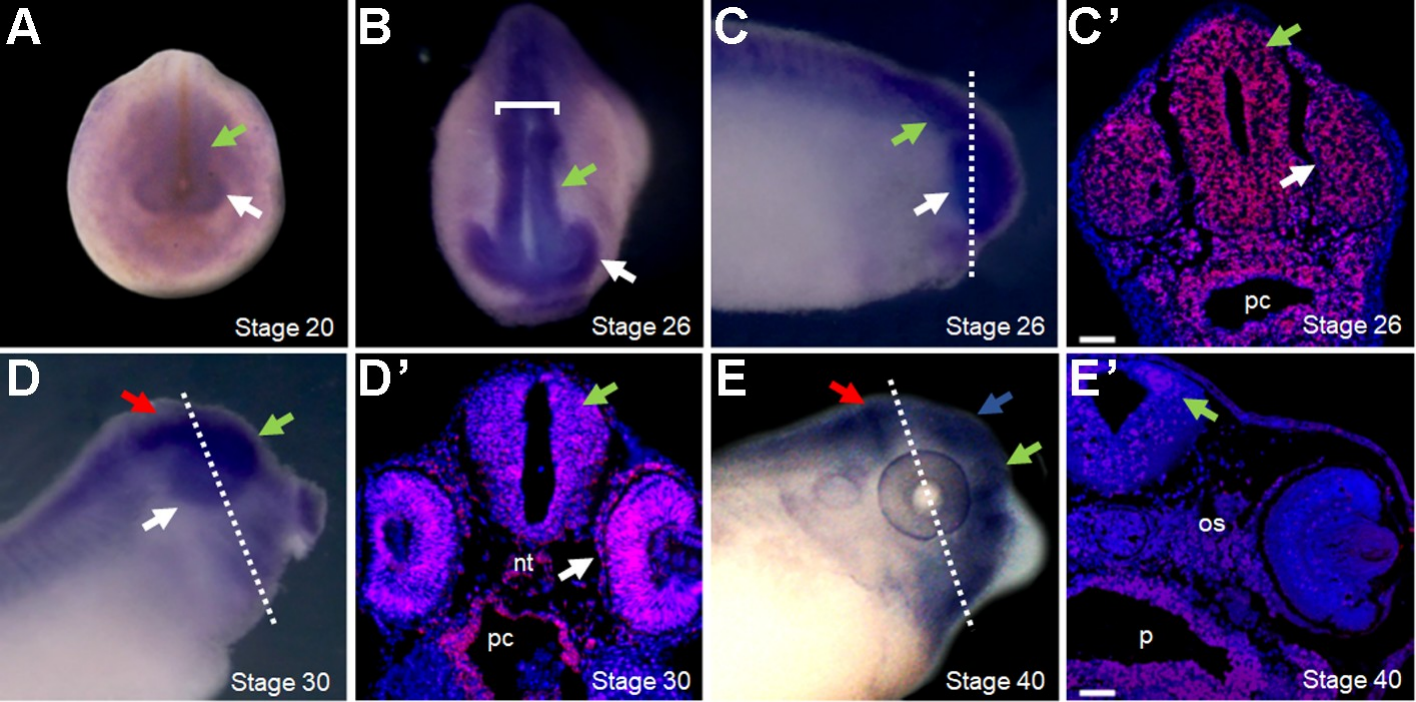
Spatiotemporal expression of *Jmjd6a* in *Xenopus laevis* development. Whole-mount *in situ* hybridization with antisense riboprobe against *Xenopus Jmjd6a* and immunohistochemistry with anti-Jmjd6 antibody were performed at indicated stages (n = 3). (A) At late neurula stage (stage 20), *Jmjd6a* is expressed broadly in the anterior neural tissue including the eye primordia (white arrow) and brain primordia (green arrow). Posterior view is shown. (B and C) At early tailbud stage (stage 26), *Jmjd6a* expression is increased in the eye primordia (white arrow), brain primordia (green arrow), and neural tube (white bracket). Anterior and lateral views are shown. (C’) In transverse section of C (white dotted line), Jmjd6 protein is detected in the brain (green arrow), optic cup (white arrow), and boundary of the pharyngeal cavity (pc) at stage 26. (D) At stage 30, *Jmjd6a* is expressed in the forebrain (green arrow), hindbrain (red arrow) and eye (white arrow). Lateral view is shown. (D’) In transverse section of D (white dotted line), Jmjd6 protein is localized in the brain (green arrow), retinal layer (white arrow), notochord (nt), and boundary of the pharyngeal cavity (pc) at stage 30. (E) At stage 40, *Jmjd6a* expression is detected mainly in the forebrain (green arrow), midbrain (blue arrow), and hindbrain (red arrow). Lateral view is shown. (E’) In transverse section of E (white dotted line), Jmjd6 protein is detected in the dorsal region of brain (green arrow), optic stalk (os), and boundary of the pharynx (p) at stage 40. Nuclei were stained with DAPI. Scale bars: 50 μm.

We first examined temporal and spatial expression patterns of *Jmjd6a* and *Jmjd6b*, which are expressed maternally and zygotically [28]. Quantitative RT-PCR demonstrated that expressions of *Jmjd6a* and *Jmjd6b* were gradually decreased during *Xenopus* early development (S1 Fig). The expression level of *Jmjd6a* was higher than that of *Jmjd6b* and changed more drastically (S1 Fig). We further analyzed *Jmjd6* expression by whole-mount *in situ* hybridization using antisense *Jmjd6* riboprobes. *Jmjd6a* was expressed broadly in the anterior neural tissues including the eye and brain primordia at stage 20 (Fig 1A). At stage 26, increased *Jmjd6a* expression was detected in the eye primordia, brain primordia, and neural tube (Fig 1B and 1C), and the elevated expression of *Jmjd6a* was maintained in the developing eye and brain at stage 30 (Fig 1D). However, *Jmjd6a* expression was restricted in the forebrain, midbrain and hindbrain at stage 40 (Fig 1E). To confirm Jmjd6 expression, immunohistochemistry was performed using anti-Jmjd6 antibody, which recognizes *Xenopus* Jmjd6, in transverse sections. Jmjd6 was detected consistently in the developing eye and brain region (Fig 1C’–1E’). Also, Jmjd6 expression was detected in the retinal layers at stage 30 (Fig 1D’). Although the expression pattern of *Jmjd6b* was similar to that of *Jmjd6a* in the developing eye and brain region, its expression was greatly decreased after stage 30 (S2 Fig).

### Jmjd6a is required for *Xenopus* eye development

**Fig 2:**
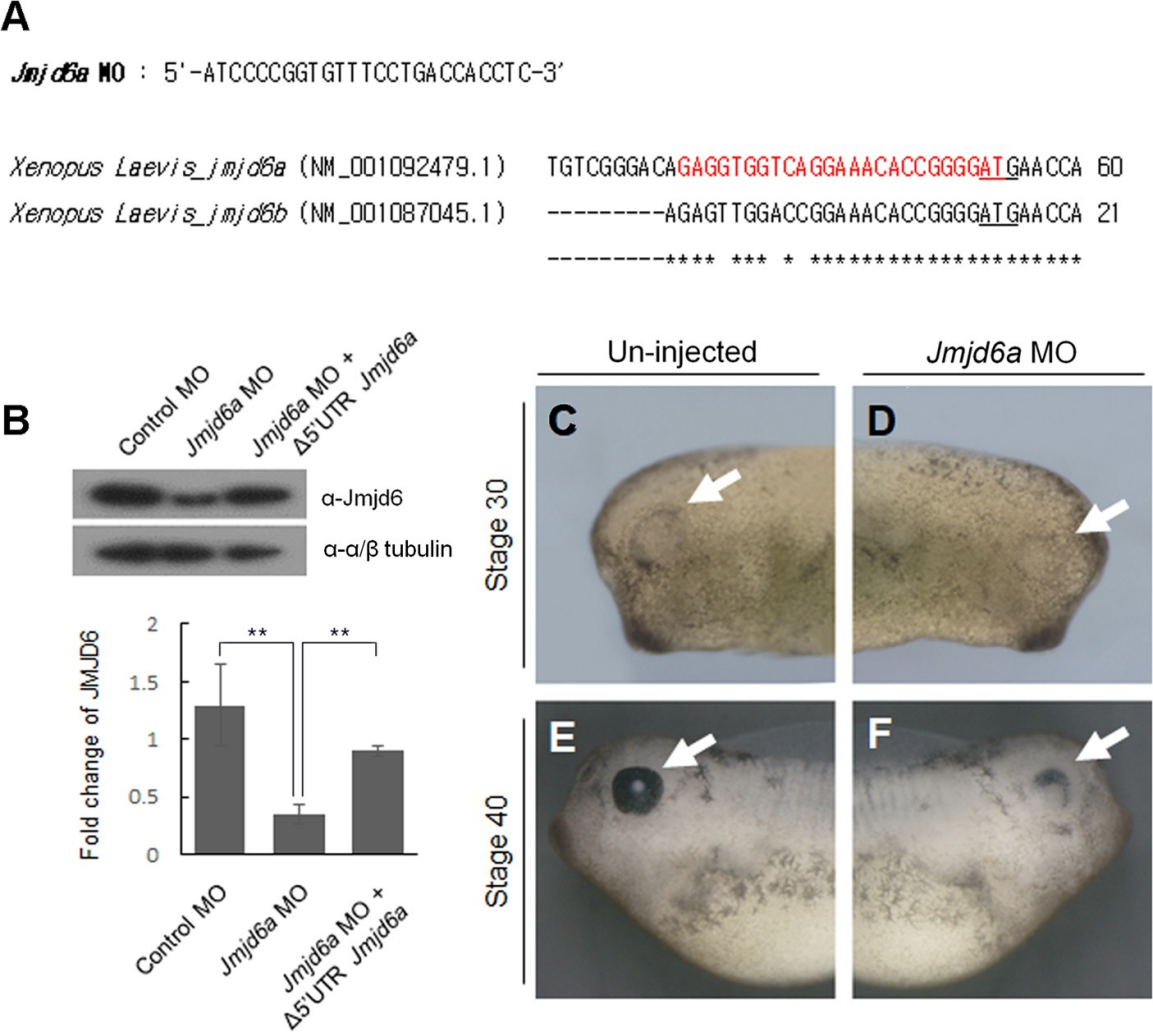
Knockdown of *Jmjd6a* affects *Xenopus laevis* eye development. (A) Antisense morpholino oligonucleotide against *Jmjd6* (*Jmjd6* MO) was designed to cover 5’ UTR and the translation start site of *Xenopus Jmjd6* gene (red). Underline denotes translation start codon. Asterisk denotes conserved nucleotide between *Xenopus Jmjd6a* and *Jmjd6b*. (B) Knockdown efficiency of *Jmjd6* MO was evaluated by western blot analysis (n = 3). To test the specificity of *Jmjd6a* MO, *Jmjd6a* mRNA without 5’UTR (Δ5’UTR *Jmjd6a*) was co-injected. Control or *Jmjd6* MO was injected into one blastomere at the 8-cell stage. Lysates from *Xenopus* embryos (stage 26) were immunoblotted with anti-Jmjd6 or anti-tubulin antibodies. Western blots were analyzed quantitatively. Data represent mean ± SD. Significance value is **P ≤ 0.01. (C~F) Compared with the un-injected side of embryos, smaller eye at stage 30 (white arrow) and partially developed eye (white arrow) were observed in *Jmjd6* MO-injected sides (n = 3). Lateral views are shown.

**Fig 3:**
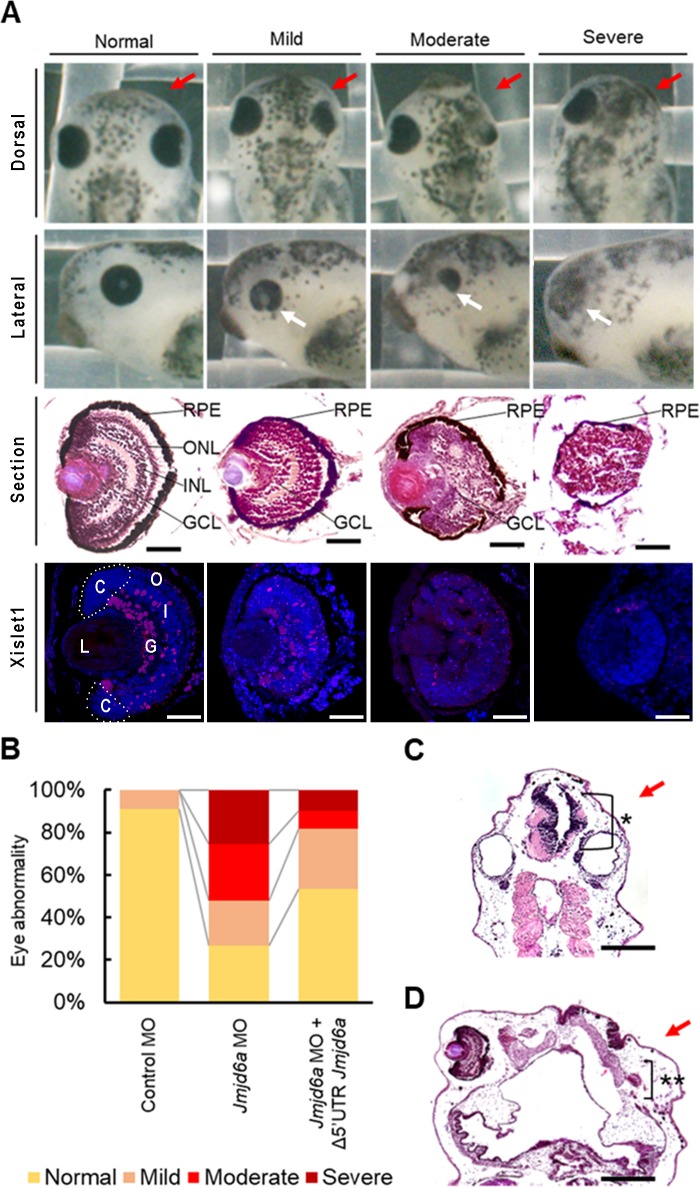
Knockdown of *Jmjd6a* leads to various eye defects in *Xenopus laevis* development. (A) Injection of *Jmjd6* MO results in various eye defects (normal to severe) in the injected side of embryos (red arrows) at stage 40 (n = 3). Dorsal (1^st^ row) and lateral (2^nd^ row) views are shown. In transverse sections (3^rd^ row), retinal layers including the RPE, ONL, INL and GCL are deformed in the *Jmjd6* MO-injected side of embryos. In severe cases, the lens is not formed. Immunohistochemical examination (4^th^ row) reveals that expression of Islet1, which is expressed in the GCL and INL in normal *Xenopus* eye, is decreased in *Jmjd6* MO injected embryos. In severe cases, expression of Islet1 is barely detected. Scale bars: 100 μm. (B) Quantification of abnormal eye phenotypes. Abnormal eye phenotypes are rescued by co-injection of *Jmjd6a* mRNA without 5’UTR (Δ5’UTR *Jmjd6a*). (C) In longitudinal section of embryo at stage 40, *Jmjd6* MO-injected sides (red arrow) show abnormal brain (*) structure. Scale bars: 20 μm. (D) In longitudinal section of embryo at stage 40, *Jmjd6* MO-injected sides (red arrow) shows formation of an ectopic eye, which developed incompletely (**) in embryos without eye formation. Scale bars: 20 μm. RPE: retinal pigment epithelium, ONL (O): outer nuclear layer, INL (I): inner nuclear layer, GCL (G): ganglion cell layer, L: lens, C: ciliary marginal zone.

To examine whether knockdown of *Jmjd6a* affects *Xenopus* eye development, we designed antisense morpholino oligonucleotide (MO) against *Xenopus Jmjd6a* (Fig 2A). Knockdown of *Jmjd6a* by MO efficiently reduced the endogenous level of Jmjd6 protein (Fig 2B). However, co-injection of *Jmjd6a* mRNA without 5’-UTR (Δ5’UTR *Jmjd6a*), which cannot be paired with *Jmjd6a* MO, restored Jmjd6 expression (Fig 2B). To examine the effect of *Jmjd6a* knockdown on eye development, MO was injected into a single animal-dorsal blastomere of 8-cell stage embryos [34]. Proper MO injection was confirmed by co-injection of plasmid containing RFP (red fluorescence protein) cDNA as a lineage tracer (S3 Fig). Knockdown of *Jmjd6a* by MO injection resulted in smaller or partially developed eyes (Fig 2C–2F). We further analyzed 468 embryos and categorized them in accordance with the extent of abnormal eye phenotypes such as the size and shape of retinal pigment epithelium (Fig 3A and 3B). Twenty one % (100/468) of embryos showed mild abnormality with smaller eye size compared to normal eyes. Twenty six % (123/468) of embryos showed moderate abnormalities with partially developed eyes. Twenty six % (120/468) of embryos had severe phenotypes including poor eye development or no eye formation. Histological observation further supported abnormal eye development. In normal developing eye, the RPE, ONL, INL, and GCL were well formed. However, some retinal layers were missing or were indistinguishable in *Jmjd6a* MO-injected embryos (Fig 3A, 3^rd^ row). In severe cases, no lens was formed (Fig 3A, 2^nd^ and 3^rd^ row). In addition, an ectopic eye, which developed incompletely, was found in embryos with no eye formation (Fig 3D). We also examined expression of Islet1, a marker of *Xenopus* retinal development, using anti-Islet1 antibody [35]. In normal developing eye, Islet1 was expressed in most of the cells in the GCL and a few cells of the INL. However, the expression level of Islet1 was decreased in defected eyes induced by *Jmjd6a* MO injection (Fig 3A, 4^th^ row). As expected, co-injection of *Jmjd6a* mRNA without 5’-UTR (Δ5’UTR *Jmjd6a*) with *Jmjd6a* MO resulted in a reduced number of embryos with abnormal eye (Fig 3B). In addition, we found malformation of anterior brain structures in *Jmjd6a* MO-injected embryos (Fig 3C).

### Knockdown of *Jmjd6a* affects expression of genes related to *Xenopus* eye development

**Fig 4:**
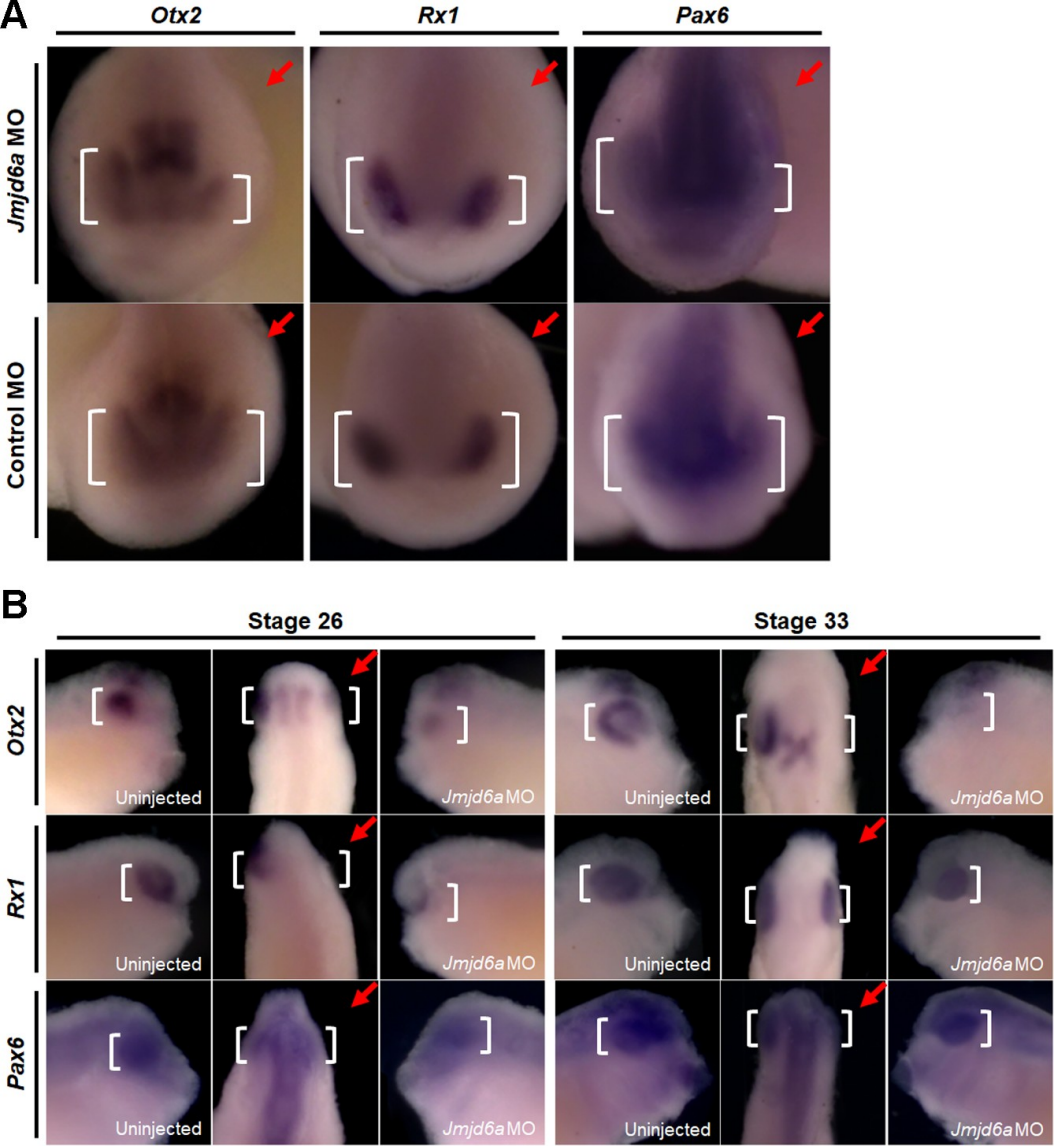
Knockdown of *Jmjd6a* reduces the expression of genes related to *Xenopus* eye development. Expression of genes related to *Xenopus* eye development was analyzed by whole-mount *in situ* hybridization (n = 3). (A) At stage 22, expressions of *Otx2*, *RX1*, and *Pax6* (white bracket) are decreased in the *Jmjd6a* MO-injected side (red arrow). However, the gene expression (white bracket) is not altered in the control MO-injected sides (red arrow) of embryos. Anterior view is shown. (B) At stages 26 and 33, the gene expression (white bracket) is also decreased in the *Jmjd6a* MO-injected side (red arrow). Lateral (left and right) and dorsal (middle) views are shown.

To investigate the effect of *Jmjd6a* MO on expression of genes related to *Xenopus* eye development, we analyzed the expression of *Otx2* (developmental marker for forebrain, eye, and anterior midbrain), *Rx1* (developmental marker for eye), and *Pax6* (developmental marker for forebrain and eye) at tailbud stages (stage 22, 26 and 33). We found that expression of *Otx2*, *Rx1*, and *Pax6* decreases in the eye primordia at stage 22 in the *Jmjd6a* MO injected side compared with the un-injected or control MO-injected side of embryos (Fig 4A). However, expression of *Otx2* and *Pax6* was not affected in the brain region (Fig 4A). At stages 26 and 33, decreased expressions of *Otx2*, *Rx1*, and *Pax6* gene were maintained (Fig 4B).

### Jmjd6a acts as RNA splicing regulator of *Xenopus GSK3β*

**Fig 5:**
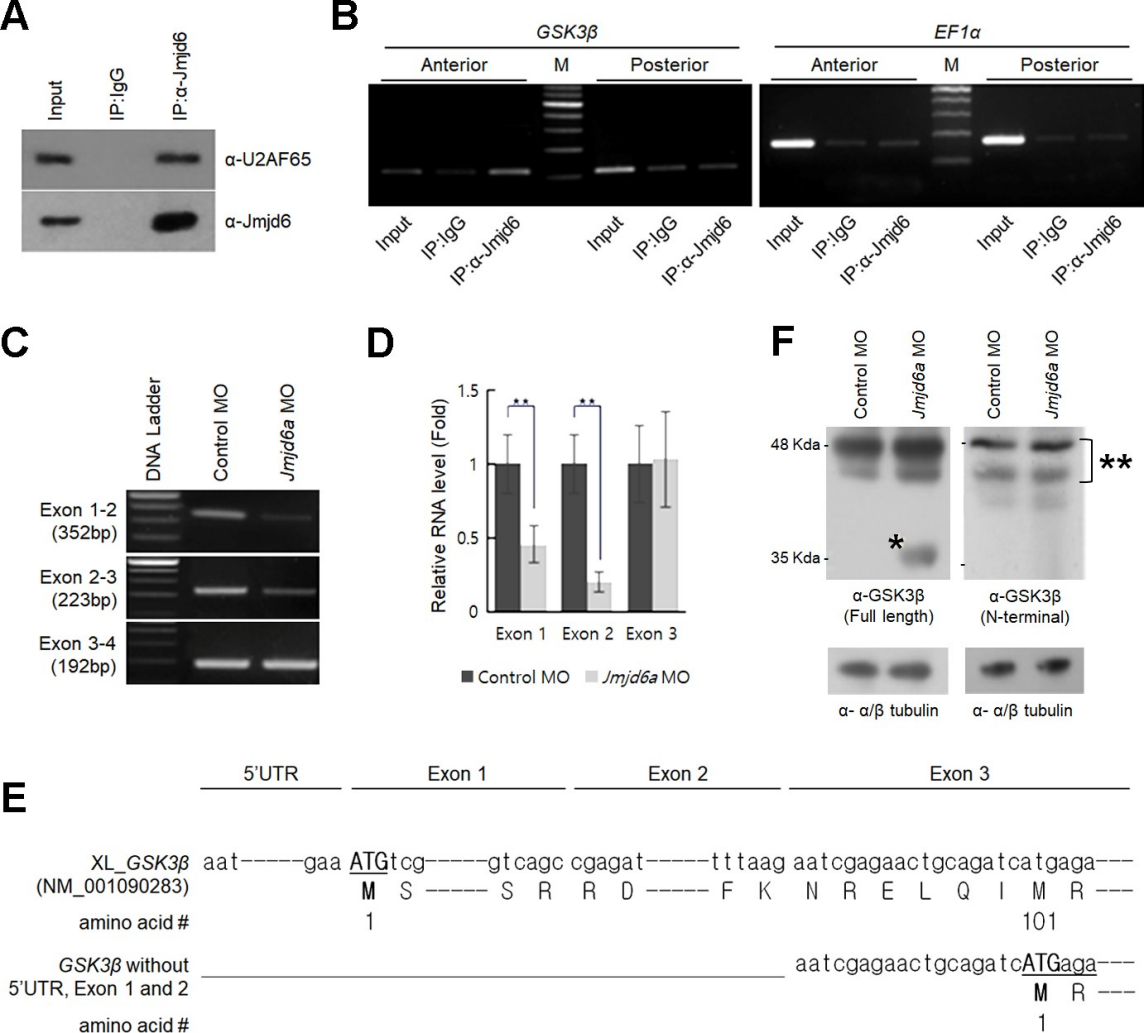
Jmjd6a regulates *GSK3β* RNA splicing in *Xenopus laevis* development. (A) Jmjd6 interacts with U2AF65. Lysate from the anterior region of embryos (stage 26) was subjected to immunoprecipitation with anti-Jmjd6 antibody, and immunoblot with anti-U2AF65 antibody (n = 3). As a negative control, IgG was used. (B) Jmjd6 interacts with *Xenopus GSK3β* RNA. RNA-immunoprecipitation was performed with Jmjd6 antibody using lysates from the anterior and posterior region of embryos (stage 26) (n = 3). *Xenopus GSK3β* and *EF1α* RNA were analyzed by RT-PCR. As a negative control, IgG was used. (C) Knockdown of *Jmjd6a* induces generation of *Xenopus GSK3β* RNA without exon 1 and 2. RT-PCR was performed in the control or *Jmjd6a* MO-injected anterior region of embryos using exon-specific oligonucleotides (n = 3). (D) Real time-PCR analysis for *Xenopus GSK3β* RNA splicing in the control or *Jmjd6a* MO-injected anterior region of embryos using exon-specific oligonucleotides (n = 3). Relative expressions were normalized with *GSK3β* RNA exon 10. Data represent mean ± SD. Significance values are **P ≤ 0.01. (E) 5’RACE (rapid amplification of cDNA ends) analysis indicates that *GSK3β* RNA starts with exon 3 in *Jmjd6a* MO-injected embryos. (F) Knockdown of *Jmjd6a* results in generation of an N’-terminal truncated form of *Xenopus* GSK3β protein with a molecular weight of approximately 35kDa (*). Endogenous full length of GSK3β proteins are denoted as **. Western blot analysis using lysate from the anterior region of embryos at stage 26 was performed with antibodies recognizing full-length or N-terminal of GSK3β (n = 3). Anti-tubulin antibody was used as a loading control.

Mammalian Jmjd6 regulates RNA splicing by interacting with specific SR-related proteins or catalyzing lysyl-hydroxylation of splicing factor U2AF65 (U2 small nuclear ribonucleoprotein auxiliary factor 65-kilodalton subunit) [19, 21, 22]. Thus, first we examined the interaction between Jmjd6 and U2AF65 by an immunoprecipitation assay using lysate from Jmjd6a expressing anterior region of embryos at stage 26. Consistently, we found that Jmjd6 interacts with *Xenopus* U2AF65 (Fig 5A). To identify the possible RNA target of Jmjd6a in *Xenopus* eye development, we searched a mouse database that presented Jmjd6-associated RNAs using an RNA-immunoprecipitation assay [22]. Among them, we chose *GSK3β* as a candidate because of its critical role in vertebrate eye development [36–38]. To confirm Jmjd6 interaction with *Xenopus GSK3β* RNA, RNA-immunoprecipitation was performed using lysate from the anterior region of *Xenopus* embryos. RT-PCR analysis indicated that Jmjd6 associates with *GSK3β* RNA (Fig 5B). However, no or weak interaction was detected in the posterior region of embryos, where the expression level of *Jmjd*6a was low. In addition, Jmjd6 showed no interaction with *EF1α* RNA, which was used as a negative control. Based on these results, we next examined whether knockdown of *Jmjd6a* affects the splicing pattern of *GSK3β* RNA, which consists of 11 exons, in developing *Xenopus* embryos. We performed RT-PCR analysis with exon specific oligonucleotides in control or *Jmjd6a* MO-injected embryos at stage 26. The *Jmjd6a* MO injection resulted in decreased levels of *GSK3β* exon 1 and 2 compared with the control MO-injected embryos (Fig 5C and 5D). However, other exons were not changed in either the control or *Jmjd6a* MO-injected embryos (S4 Fig). We confirmed the generation of *GSK3β* RNA without exon 1 and 2 by 5’ RACE (rapid amplification of cDNA ends) analysis in *Jmjd6a* MO-injected embryos at stage 26 (Fig 5E). The aberrant *GSK3β* RNA started with the exon 3 containing ATG start codon (Fig 5E). Because the absence of *GSK3β* exon 1 and exon 2 is expected to result in the loss of 94 N’-terminal amino acids of the protein, we next performed western blot analysis using a lysate from *Xenopus* embryos injected with *GSK3β* mRNA started with exon 3. We found an extra 35 kDa band of GSK3β protein as well as endogenous full length proteins (S5 Fig). Consistently, knockdown of *Jmjd6a* by MO injection resulted in the production of a 35 kDa band of GSK3β protein in *Xenopus* embryos, with no such band being detected in control MO injected embryos (Fig 5F). Moreover, an extra 35 kDa band was not detected in western blot analysis with anti-GSK3β antibody recognizing N’-terminal protein (Fig 5F). These results may suggest that Jmjd6a interacts with splicing factor U2AF65 and regulates *GSK3β* RNA splicing in *Xenopus* eye development.

### Knockdown of *Jmjd6a* affects canonical Wnt/β-catenin signaling in *Xenopus laevis* development

**Fig 6:**
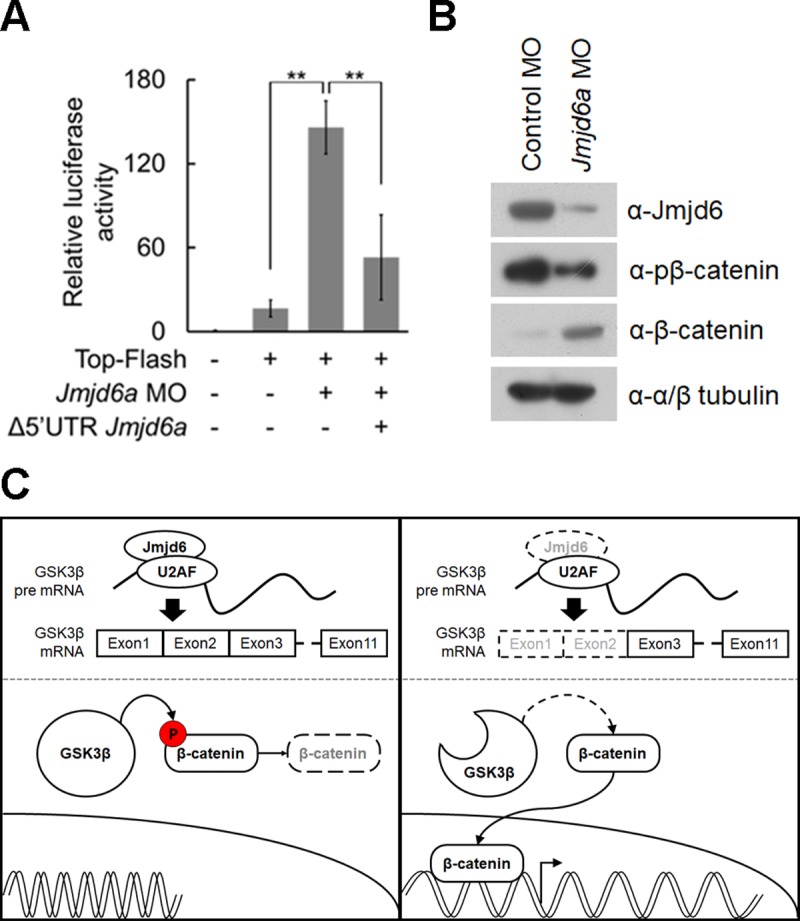
Altered splicing of GSK3β by *Jmjd6*a knockdown augments canonical Wnt/β-catenin signaling in *Xenopus laevis* development. (A) Knockdown of *Jmjd6a* activates canonical Wnt/β-catenin signaling. TOP-Flash plasmid containing TCF binding sites was co-injected with control or *Jmjd6a* MO into *Xenopus* embryos at stage 26 (n = 3). Firefly luciferase assay was performed using lysate from the anterior region of injected embryos. Renilla luciferase activity was used to normalize firefly luciferase. Over-expression of *Jmjd6a* (Δ5’UTR *Jmjd6a*) suppresses up-regulation of TOP-flash activity in *Jmjd6a* MO-injected embryos. Data represent mean ± SD. Significance values are **P ≤ 0.01. (B) Knockdown of *Jmjd6a* induces decreased phosphorylation of β-catenin and increased stability of β-catenin. Lysates from the anterior region of the control or *Jmjd6a* MO-injected embryos (stage 26) were immunoblotted with anti-Jmjd6, anti-β-catenin, and anti-phospho β-catenin (Ser33, Ser37, Thr41) antibodies (n = 3). Anti-Tubulin antibody was used as a loading control. (C) Proposed model for Jmjd6a-mediated regulation of canonical Wnt/β-catenin signaling in *Xenopus* eye development.

Given that Jmjd6a regulates *GSK3β* RNA splicing, we next investigated the effect of *Jmjd6a* knockdown on canonical Wnt/β-catenin signaling using a TOP-flash luciferase reporter containing multiple TCF binding sites. We injected the TOP-flash reporter with *Jmjd6a* MO into *Xenopus* embryos at stage 26. Although basal activity of luciferase was observed in the TOP-flash reporter-injected embryos, *Jmjd6*a MO co-injection resulted in an increase of luciferase activity (Fig 6A). However, co-injection of *Jmjd6a* mRNA without 5’-UTR (Δ5’UTR *Jmjd6a*) suppressed increased luciferase activity by *Jmjd6a* knockdown. Consistently, phosphorylation of β-catenin was decreased and stability of β-catenin was increased in *Jmjd6a* MO-injected embryos (Fig 6B). Taken together, our results demonstrate that knockdown of *Jmjd6a* results in aberrant *GSK3β* RNA splicing and generation of an N-terminal truncated form of GSK3β protein, which induces increased β-catenin stability.

## Discussion

The developmental process of eukaryotes requires tightly regulated gene expression at multiple levels including RNA splicing. It has been reported that mammalian Jmjd6 regulates gene expression by modification of histones or transcription factors [13, 18, 39]. Jmjd6 also regulates RNA splicing by interaction of splicing factors [19, 21, 40]. We and others found that two pseudoalleles of *Jmjd6*, *Jmjd6a* and *Jmjd6b*, are expressed in developing *Xenopus* eye, brain, and neural tube [28]. Immunohistochemical examination demonstarted that the expression pattern of Jmjd6 ptrotein was similar to that of *Jmjd6* RNA, and Jmjd6 protein was also expressed in retinal layers at stage 30. Similar spatiotemporal expression of *Jmjd6* has been reported in developing mouse embryos [29]. These results suggest that Jmjd6 may play an important role in *Xenopus* eye developmental processes such as optic vesicle formation and retinal layer differentiation.

In this study, we found that knockdown of *Jmjd6a* induces abnormal eye development with mild to severe abnormalities. Histological examination further revealed deformed retinal layer formation as well as no lens formation. Immunohistochemical examination of Islet1, a marker of *Xenopus* retina development, showed aberrant retinal cell differentiation in *Jmjd6a* MO injected embryos. Islet1 is expressed in ganglion, amacrine, bipolar, and horizontal cells in the GCL and INL [35]. However, decreased expression of Islet1 was detected in *Jmjd6a* MO injected embryos. Consistent with our result, *Jmjd6* knockout embryos showed eye malformations that ranged from abnormal differentiation in retinal cell layers including the INL to anophthalmia (no eye formation). Moreover, ectopic eyes were found in case of anophthalmia [29]. We further found that knockdown of *Jmjd6a* altered gene expression related to eye development including *Otx2*, *Rx1*, and *Pax6*. *Otx2* is expressed in the entire optic vesicle at the initial stage of eye development, and its expression is restricted to the presumptive RPE at later stages [41]. *Rax*, which is a mouse homolog of *Rx1*, is intensively expressed in the developing retina in mouse [42] and the ONL of *Xenopus* eyes [43]. *Pax6* is an important transcription factor for eye development because ectopic expression of *Pax6* alone is sufficient to induce ectopic eyes in fly and frog embryos [44, 45]. *Pax6* is expressed highly in the early optic vesicles and the surface ectoderm, and its expression remains in all eye components at the optic-cup stage. Its expression becomes restricted to the lens, corneal and conjunctive epithelia, iris, and inner portion of the neuroretina [46].

Previous studies have shown that Jmjd6 regulates RNA splicing by interaction with splicing factors [19, 22, 40]. We confirmed the interaction of Jmjd6 with U2AF65 splicing factor and *GSK3β* RNA in *Xenopus* embryos. Knockdown of *Jmjd6a* resulted in *GSK3β* RNA lacking exon 1 and 2, thereby generating an N’-terminal truncated form of GSK3β protein with a molecular weight of approximately 35 kDa. A few studies have been described the importance of the N’-terminal region of GSK3β, for instance the N’-terminal of GSK3β may regulate its kinase activity on β-catenin because of the presence of critical lysine resides (K85 and K86) located at the ATP binding site of GSK3β [47–49]. Moreover, kinase dead mutant of GSK3β (GSK3β K85M) has been shown to abolish the interaction with axin, indicating that kinase actitivy is required for β-catenin destruction [50, 51]. Thus, generation of an N’-terminal truncated form of GSK3β protein induced by the knockdown of *Jmjd6a* may induce decreased β-catenin phosphorylation and consequently increased β-catenin stability.

It is well known that canonical Wnt/β-catenin signaling pathway plays a critical role in vertebrate eye development. For instance, canonical Wnt/β-catenin signaling is active in the dorsal optic vesicle and presumptive RPE at the optic vesicle stage. It is subsequently restricted to the peripheral RPE [5, 52, 53]. At the optic cup stage, RPE transdifferentiates into the neural retina in the absence of β-catenin [53, 54]. Mis-regulation of canonical Wnt/β-catenin signaling results in multiple eye malformations because of defects in the process of cell fate determination and differentiation [6]. Consistent with our results demonstrating increased level of β-catenin protein by *Jmjd6a* knockdown, overexpression of constitutively active β-catenin results in disorganization of the retina layers in mouse [53, 55].

In our model, Jmjd6a and U2AF65 bind to *GSK3β* RNA, resulting in inclusion of exon 1 and 2. Subsequently, GSK3β phosphorylates β-catenin and β-catenin is degraded during *Xenopus* optic vesicle formation and retinal cell differentiation (Fig 6C). However, knockdown of *Jmjd6a* results in loss of *GSK3β* RNA exon 1 and 2, thereby generating an N’-terminal truncated form of GSK3β protein. In turn, the truncated form of GSK3β may not phosphorylate β-catenin, leading to increased β-catenin stability (Fig 6C). These events further result in abnormal development of eye as well as of brain. In this study, we demonstrated Jmjd6a-mediated RNA splicing of GSK3β, which is highly a conserved kinase for cellular signaling pathways such as PI3 kinase, Wnt, Hedgehog, and Notch signaling in embryonic development, cellular differentiation, and several human diseases [56, 57]. Therefore, our findings may expand our knowledge of the functions of Jmjd6 in animal development as well as multiple human diseases including cancer [58].

## Supporting information

S1 Fig Temporal *Jmjd6a* and *b* gene expression in *Xenopus laevis* development. Quantitative RT-PCR was performed using whole *Xenopus* embryos from 8cell stage to stage 30. Data represent mean ±SD. Significance values were *P ≤ 0.05 and **P ≤ 0.01.
(TIF)

S2 Fig Spatiotemporal expression of *Jmjd6b* in *Xenopus laevis* development. Whole-mount *in situ* hybridization of *Jmjd6b* was performed at indicated stages (n = 3). (A) At late neurula stage (stage 20), *Jmjd6b* is expressed in the eye primordia (white arrow), brain primordia (green arrow), and neural tube (red arrow). Posterior view is shown. (B) At early tailbud stage (stage 25), *Jmjd6b* expression is detected in the eye (white arrow) and brain region (red arrow). Lateral view is shown. (C) At stage 30, *Jmjd6b* is expressed in the eye (white arrow). Lateral view is shown. (D) At stage 40, *Jmjd6b* expression is not detected. Lateral view is shown.
(TIF)

S3 Fig Confirmation of MO injection. To confirm proper MO injection, plasmid containing RFP (red fluorescence protein) cDNA was co-injected into one blastomere at the 8-cell stage. (A) Lateral view of un-injected side of embryo. (B) Dorsal view of embryo. RFP-injected side of embryo is shown to the right. (C) Lateral view of RFP-injected side of embryo. Note the red fluorescence in RFP-injected side of embryo.
(TIF)

S4 Fig Splicing pattern of *Xenopus GSK3β* in *Jmjd6a* MO-injected embryos. Each *GSK3β* exon (4~11) was amplified by real time-PCR in the anterior region of *Jmjd6a* MO-injected embryos (stage 26) using exon specific oligonucleotides. Relative expressions were normalized with *GSK3β* RNA exon 10 because its expression was not changed based on *EF1a* expression. Data represent mean ± SD (Exon 3, *p = 0*.*22*; Exon 4, *p = 0*.*25*; Exon 5, *p = 0*.*11*; Exon 6, *p = 0*.*12*; Exon 7, *p = 0*.*15*; Exon 8, *p = 0*.*55*; Exon 9, *p = 0*.*13*; Exon 11, *p = 0*.*39*).
(TIF)

S5 Fig Translation of N-terminal truncated form of *Xenopus* GSK3β. Synthesized *GSK3β* RNA without exon1 and 2 was injected into *Xenopus* embryos and the lysate was analyzed by western blotting using an antibody recognizing the full length of GSK3β. An extra 35 kDa band of GSK3β is detected (*). Endogenous full length of GSK3β is denoted as **.
(TIF)

S1 Table Oligonucleotides used in this study. (PPTX)

S1 File Raw values for statistical analysis. (XLSX)
