# Supplementary figures and images for: Jmjd6a regulates GSK3β RNA splicing in Xenopus laevis eye development
Source: PLoS One. 2019 Jul 30;14(7):e0219800. doi: 10.1371/journal.pone.0219800 (PMC6667200; doi:10.1371/journal.pone.0219800)

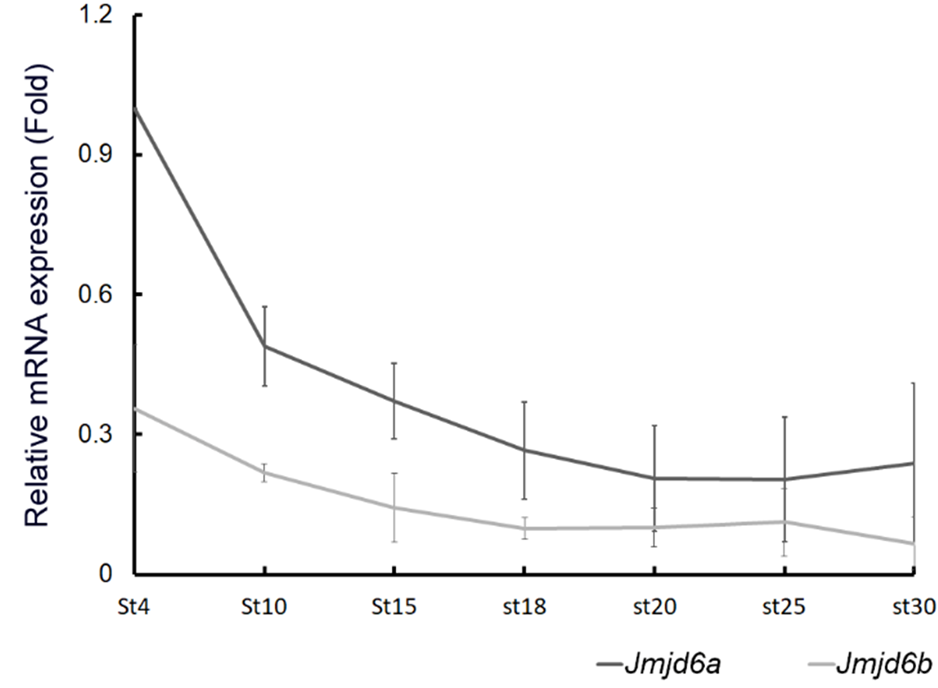

Supplement: S1 Fig — Quantitative RT-PCR was performed using whole Xenopus embryos from 8cell stage to stage 30. Data represent mean ±SD. Significance values were *P ≤ 0.05 and **P ≤ 0.01. (TIF) [file pone.0219800.s001.tif]

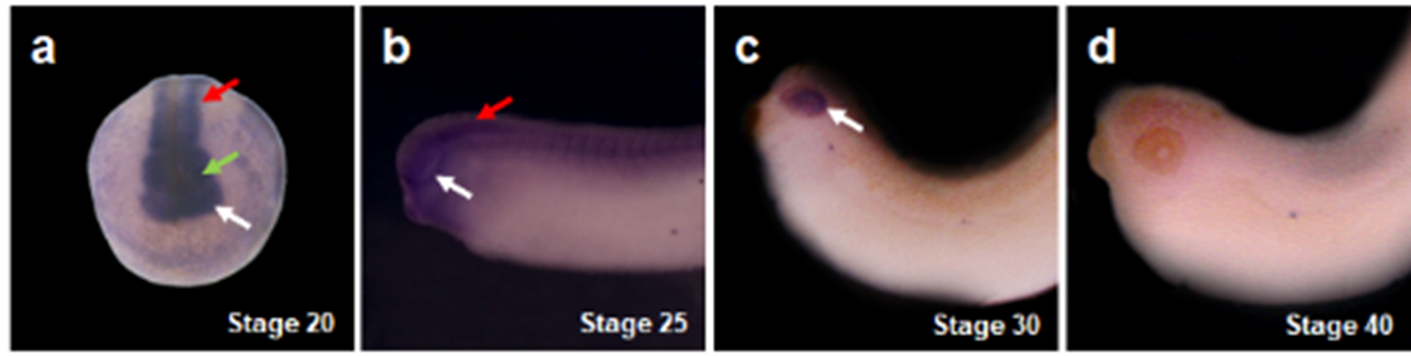

Supplement: S2 Fig — Whole-mount in situ hybridization of Jmjd6b was performed at indicated stages (n = 3). (A) At late neurula stage (stage 20), Jmjd6b is expressed in the eye primordia (white arrow), brain primordia (green arrow), and neural tube (red arrow). Posterior view is shown. (B) At early tailbud stage (stage 25), Jmjd6b expression is detected in the eye (white arrow) and brain region (red arrow). Lateral view is shown. (C) At stage 30, Jmjd6b is expressed in the eye (white arrow). Lateral view is shown. (D) At stage 40, Jmjd6b expression is not detected. Lateral view is shown. (TIF) [file pone.0219800.s002.tif]

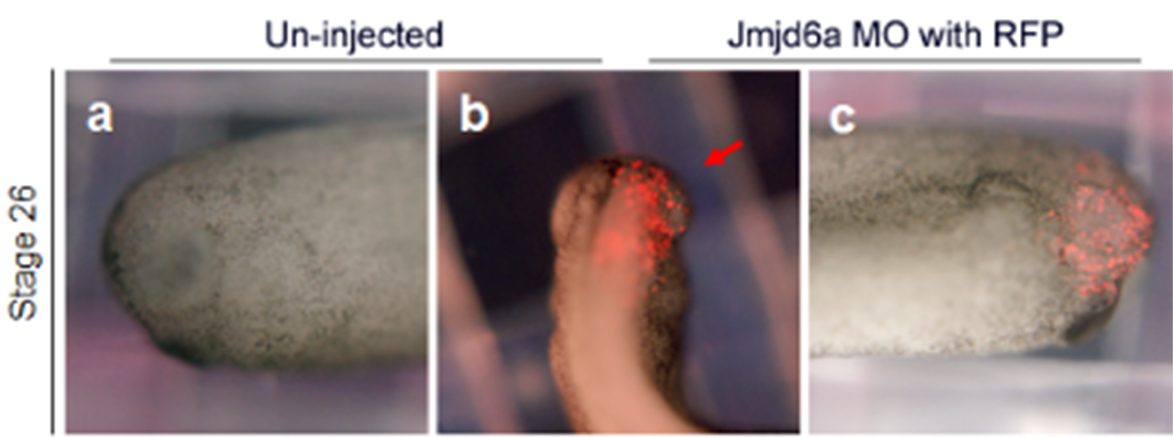

Supplement: S3 Fig — To confirm proper MO injection, plasmid containing RFP (red fluorescence protein) cDNA was co-injected into one blastomere at the 8-cell stage. (A) Lateral view of un-injected side of embryo. (B) Dorsal view of embryo. RFP-injected side of embryo is shown to the right. (C) Lateral view of RFP-injected side of embryo. Note the red fluorescence in RFP-injected side of embryo. (TIF) [file pone.0219800.s003.tif]

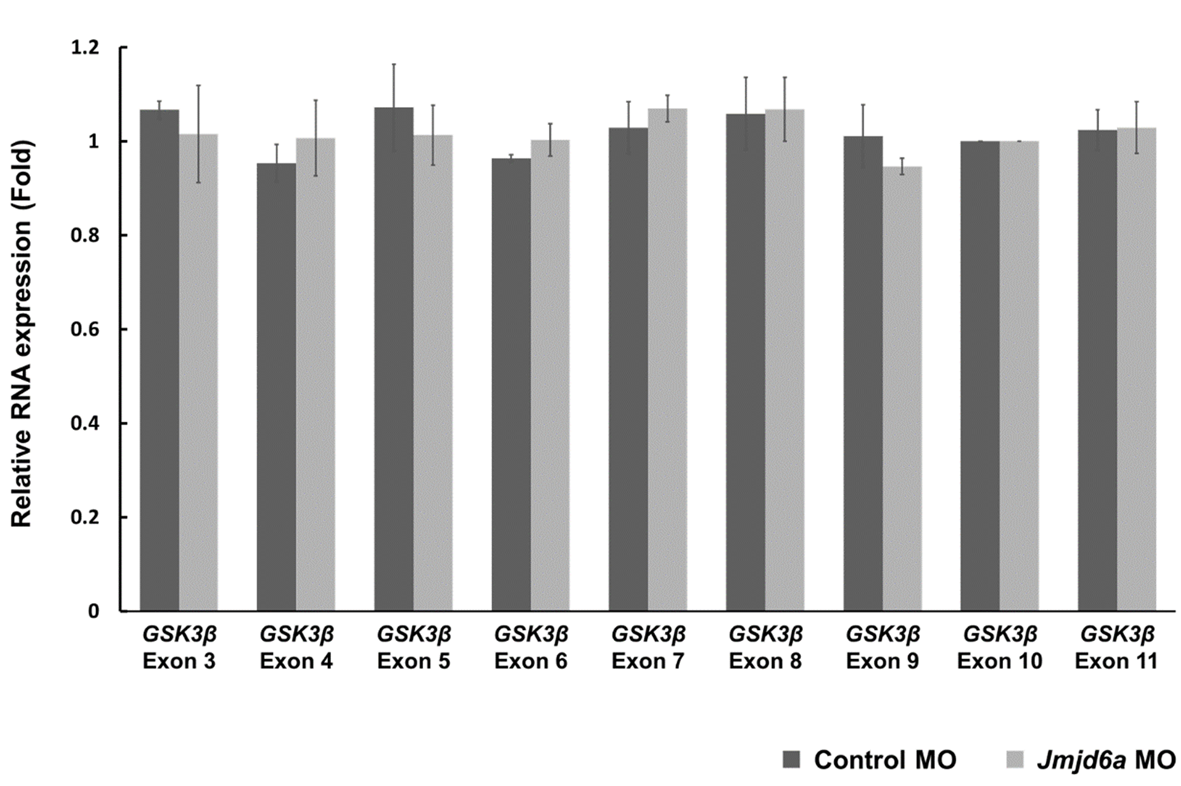

Supplement: S4 Fig — Each GSK3β exon (4~11) was amplified by real time-PCR in the anterior region of Jmjd6a MO-injected embryos (stage 26) using exon specific oligonucleotides. Relative expressions were normalized with GSK3β RNA exon 10 because its expression was not changed based on EF1a expression. Data represent mean ± SD (Exon 3, p = 0.22; Exon 4, p = 0.25; Exon 5, p = 0.11; Exon 6, p = 0.12; Exon 7, p = 0.15; Exon 8, p = 0.55; Exon 9, p = 0.13; Exon 11, p = 0.39). (TIF) [file pone.0219800.s004.tif]

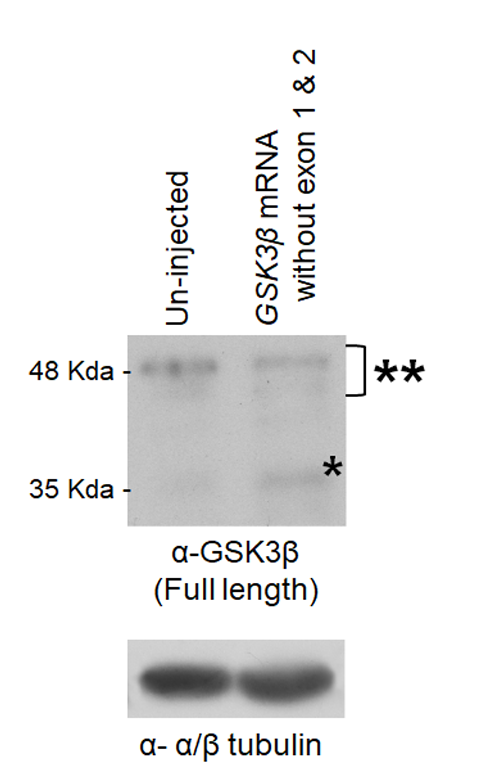

Supplement: S5 Fig — Synthesized GSK3β RNA without exon1 and 2 was injected into Xenopus embryos and the lysate was analyzed by western blotting using an antibody recognizing the full length of GSK3β. An extra 35 kDa band of GSK3β is detected (*). Endogenous full length of GSK3β is denoted as **. (TIF) [file pone.0219800.s005.tif]
