# Supplementary material for: Jmjd6a regulates GSK3β RNA splicing in Xenopus laevis eye development
Source: PLoS One. 2019 Jul 30;14(7):e0219800. doi: 10.1371/journal.pone.0219800 (PMC6667200; doi:10.1371/journal.pone.0219800)
Supplement: S1 Table — (PPTX) [file pone.0219800.s006.pptx]

## Slide 1
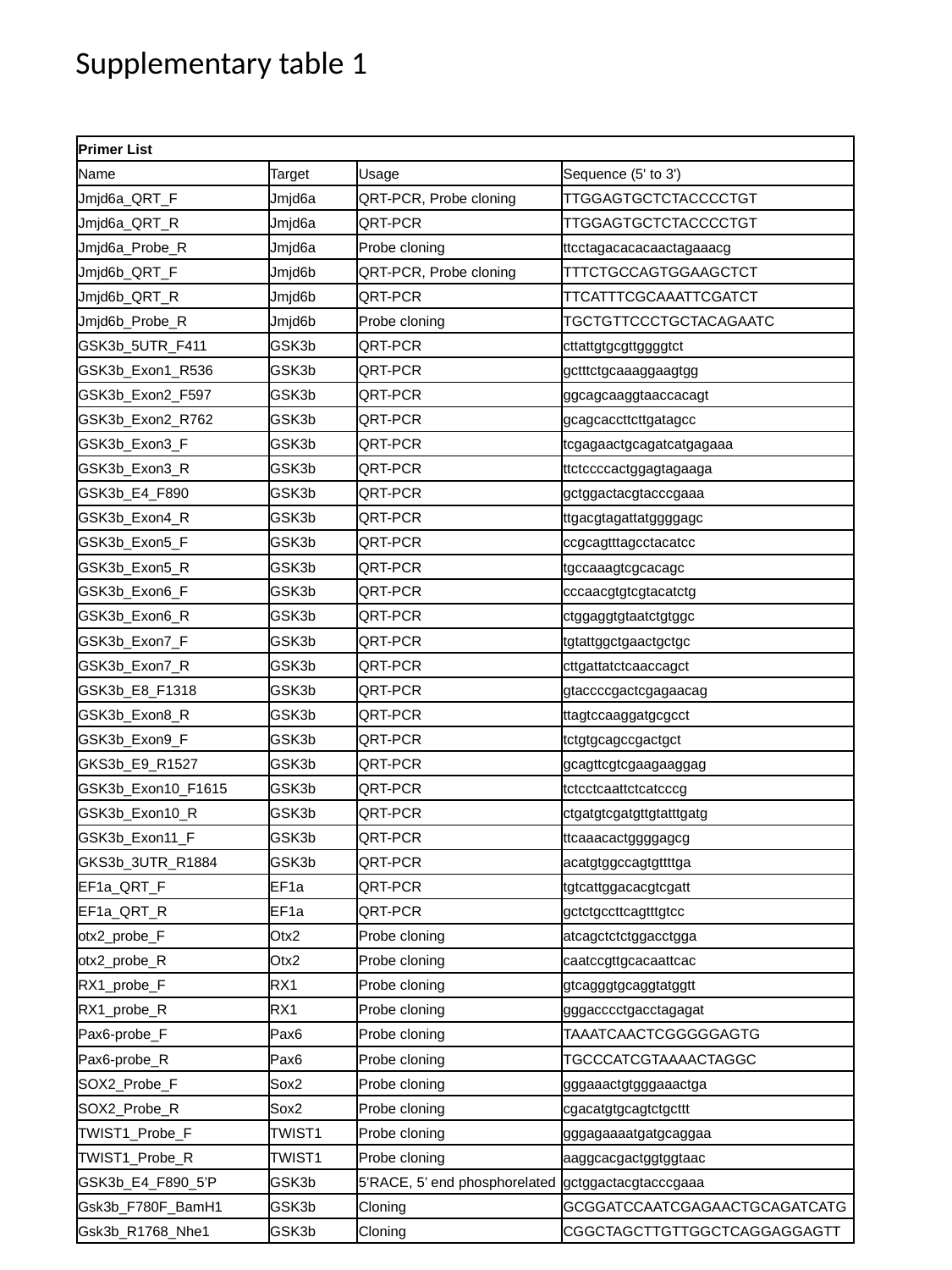

Supplementary table 1
| Primer List | | | |
| --- | --- | --- | --- |
| Name | Target | Usage | Sequence (5' to 3') |
| Jmjd6a\_QRT\_F | Jmjd6a | QRT-PCR, Probe cloning | TTGGAGTGCTCTACCCCTGT |
| Jmjd6a\_QRT\_R | Jmjd6a | QRT-PCR | TTGGAGTGCTCTACCCCTGT |
| Jmjd6a\_Probe\_R | Jmjd6a | Probe cloning | ttcctagacacacaactagaaacg |
| Jmjd6b\_QRT\_F | Jmjd6b | QRT-PCR, Probe cloning | TTTCTGCCAGTGGAAGCTCT |
| Jmjd6b\_QRT\_R | Jmjd6b | QRT-PCR | TTCATTTCGCAAATTCGATCT |
| Jmjd6b\_Probe\_R | Jmjd6b | Probe cloning | TGCTGTTCCCTGCTACAGAATC |
| GSK3b\_5UTR\_F411 | GSK3b | QRT-PCR | cttattgtgcgttggggtct |
| GSK3b\_Exon1\_R536 | GSK3b | QRT-PCR | gctttctgcaaaggaagtgg |
| GSK3b\_Exon2\_F597 | GSK3b | QRT-PCR | ggcagcaaggtaaccacagt |
| GSK3b\_Exon2\_R762 | GSK3b | QRT-PCR | gcagcaccttcttgatagcc |
| GSK3b\_Exon3\_F | GSK3b | QRT-PCR | tcgagaactgcagatcatgagaaa |
| GSK3b\_Exon3\_R | GSK3b | QRT-PCR | ttctccccactggagtagaaga |
| GSK3b\_E4\_F890 | GSK3b | QRT-PCR | gctggactacgtacccgaaa |
| GSK3b\_Exon4\_R | GSK3b | QRT-PCR | ttgacgtagattatggggagc |
| GSK3b\_Exon5\_F | GSK3b | QRT-PCR | ccgcagtttagcctacatcc |
| GSK3b\_Exon5\_R | GSK3b | QRT-PCR | tgccaaagtcgcacagc |
| GSK3b\_Exon6\_F | GSK3b | QRT-PCR | cccaacgtgtcgtacatctg |
| GSK3b\_Exon6\_R | GSK3b | QRT-PCR | ctggaggtgtaatctgtggc |
| GSK3b\_Exon7\_F | GSK3b | QRT-PCR | tgtattggctgaactgctgc |
| GSK3b\_Exon7\_R | GSK3b | QRT-PCR | cttgattatctcaaccagct |
| GSK3b\_E8\_F1318 | GSK3b | QRT-PCR | gtaccccgactcgagaacag |
| GSK3b\_Exon8\_R | GSK3b | QRT-PCR | ttagtccaaggatgcgcct |
| GSK3b\_Exon9\_F | GSK3b | QRT-PCR | tctgtgcagccgactgct |
| GKS3b\_E9\_R1527 | GSK3b | QRT-PCR | gcagttcgtcgaagaaggag |
| GSK3b\_Exon10\_F1615 | GSK3b | QRT-PCR | tctcctcaattctcatcccg |
| GSK3b\_Exon10\_R | GSK3b | QRT-PCR | ctgatgtcgatgttgtatttgatg |
| GSK3b\_Exon11\_F | GSK3b | QRT-PCR | ttcaaacactggggagcg |
| GKS3b\_3UTR\_R1884 | GSK3b | QRT-PCR | acatgtggccagtgttttga |
| EF1a\_QRT\_F | EF1a | QRT-PCR | tgtcattggacacgtcgatt |
| EF1a\_QRT\_R | EF1a | QRT-PCR | gctctgccttcagtttgtcc |
| otx2\_probe\_F | Otx2 | Probe cloning | atcagctctctggacctgga |
| otx2\_probe\_R | Otx2 | Probe cloning | caatccgttgcacaattcac |
| RX1\_probe\_F | RX1 | Probe cloning | gtcagggtgcaggtatggtt |
| RX1\_probe\_R | RX1 | Probe cloning | gggacccctgacctagagat |
| Pax6-probe\_F | Pax6 | Probe cloning | TAAATCAACTCGGGGGAGTG |
| Pax6-probe\_R | Pax6 | Probe cloning | TGCCCATCGTAAAACTAGGC |
| SOX2\_Probe\_F | Sox2 | Probe cloning | gggaaactgtgggaaactga |
| SOX2\_Probe\_R | Sox2 | Probe cloning | cgacatgtgcagtctgcttt |
| TWIST1\_Probe\_F | TWIST1 | Probe cloning | gggagaaaatgatgcaggaa |
| TWIST1\_Probe\_R | TWIST1 | Probe cloning | aaggcacgactggtggtaac |
| GSK3b\_E4\_F890\_5’P | GSK3b | 5’RACE, 5’ end phosphorelated | gctggactacgtacccgaaa |
| Gsk3b\_F780F\_BamH1 | GSK3b | Cloning | GCGGATCCAATCGAGAACTGCAGATCATG |
| Gsk3b\_R1768\_Nhe1 | GSK3b | Cloning | CGGCTAGCTTGTTGGCTCAGGAGGAGTT |
